# A narrative review of exercise participation among adults with prediabetes or type 2 diabetes: barriers and solutions

**DOI:** 10.3389/fcdhc.2023.1218692

**Published:** 2023-08-30

**Authors:** Samantha C. Thielen, Jane E. B. Reusch, Judith G. Regensteiner

**Affiliations:** ^1^ Department of Medicine, University of Colorado School of Medicine, Aurora, CO, United States; ^2^ Ludeman Family Center for Women’s Health Research, Department of Medicine, University of Colorado School of Medicine, Aurora, CO, United States; ^3^ Division of Endocrinology, Department of Medicine, University of Colorado School of Medicine, Aurora, CO, United States; ^4^ Rocky Mountain Regional Department of Veterans Affairs Medical Center (VAMC), Aurora, CO, United States; ^5^ Division of General Internal Medicine, Department of Medicine, University of Colorado School of Medicine, Aurora, CO, United States; ^6^ Division of Cardiology, Department of Medicine, University of Colorado School of Medicine, Aurora, CO, United States

**Keywords:** type 2 diabetes, exercise, barriers, cardiorespiratory fitness, prediabetes, solutions

## Abstract

Type 2 diabetes (T2D) has been rising in prevalence over the past few decades in the US and worldwide. T2D contributes to significant morbidity and premature mortality, primarily due to cardiovascular disease (CVD). Exercise is a major cornerstone of therapy for T2D as a result of its positive effects on glycemic control, blood pressure, weight loss and cardiovascular risk as well as other measures of health. However, studies show that a majority of people with T2D do not exercise regularly. The reasons given as to why exercise goals are not met are varied and include physiological, psychological, social, cultural and environmental barriers to exercise. One potential cause of inactivity in people with T2D is impaired cardiorespiratory fitness, even in the absence of clinically evident complications. The exercise impairment, although present in both sexes, is greater in women than men with T2D. Women with T2D also experience greater perceived exertion with exercise than their counterparts without diabetes. These physiological barriers are in addition to constructed societal barriers including cultural expectations of bearing the burden of childrearing for women and in some cultures, having limited access to exercise because of additional cultural expectations. People at risk for and with diabetes more commonly experience unfavorable social determinants of health (SDOH) than people without diabetes, represented by neighborhood deprivation. Neighborhood deprivation measures lack of resources in an area influencing socioeconomic status including many SDOH such as income, housing conditions, living environment, education and employment. Higher indices of neighborhood deprivation have been associated with increased risk of all-cause, cardiovascular and cancer related mortality. Unfavorable SDOH is also associated with obesity and lower levels of physical activity. Ideally regular physical activity should be incorporated into all communities as part of a productive and healthy lifestyle. One potential solution to improve access to physical activity is designing and building environments with increased walkability, greenspace and safe recreational areas. Other potential solutions include the use of continuous glucose monitors as real-time feedback tools aimed to increase motivation for physical activity, counseling aimed at improving self-efficacy towards exercise and even acquiring a dog to increase walking time. In this narrative review, we aim to examine some traditional and novel barriers to exercise, as well as present evidence on novel interventions or solutions to overcome barriers to increase exercise and physical activity in all people with prediabetes and T2D.

## 1 Introduction

**Figure 1:**
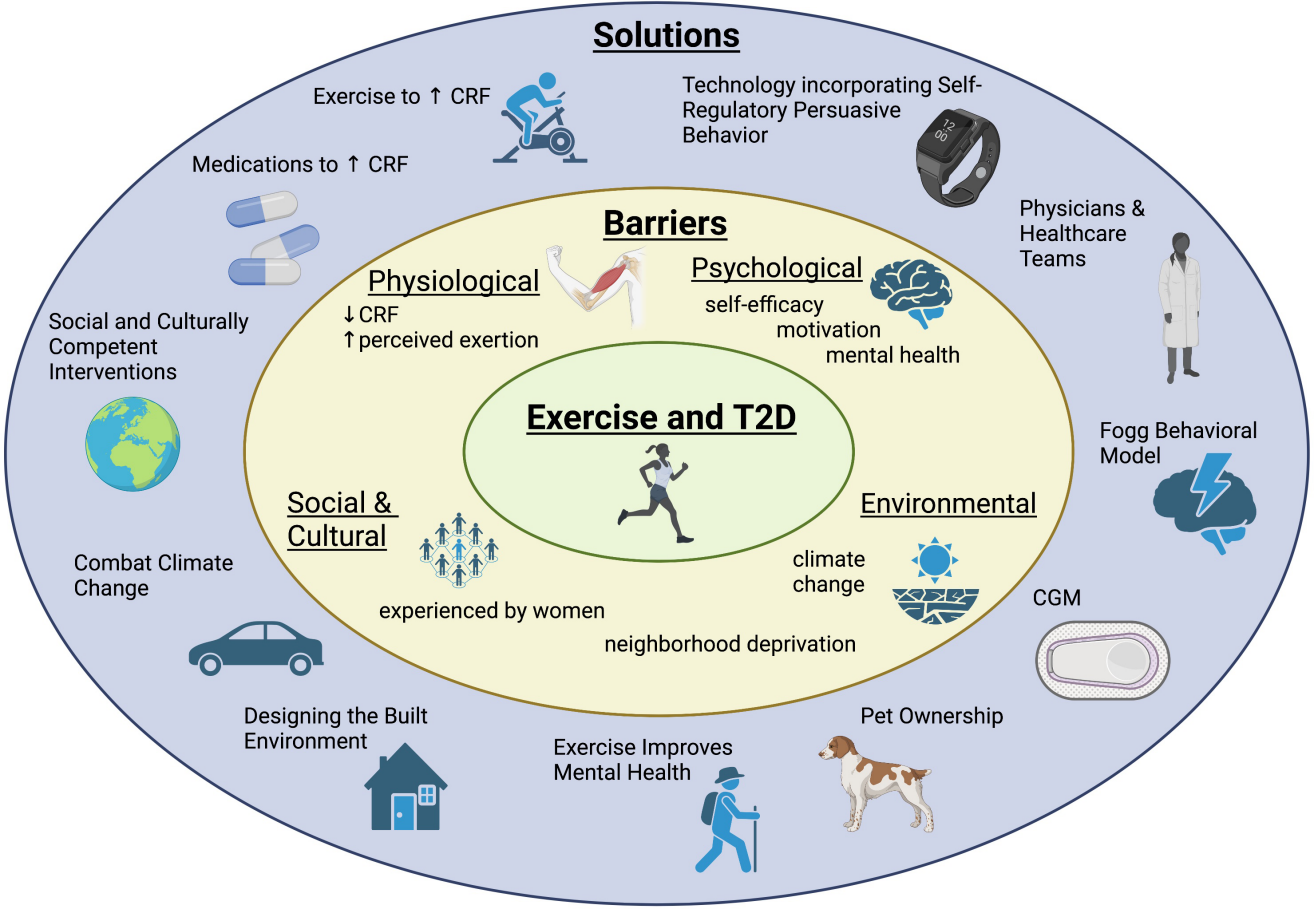
Adults with prediabetes and type 2 diabetes face a variety of barriers to exercise. This diagram highlights a summary of those barriers as well as novel interventions and solutions to overcome obstacles and increase exercise for adults with prediabetes and type 2 diabetes.

The global prevalence of type 2 diabetes (T2D) is rising, increasing from 6.2% in 2007 to 10.5% in 2021. Prevalence is predicted to continue to rise to an estimated 12.2% by the year 2045 (1, 2). This increasing prevalence is problematic since T2D confers a 2-4 times increased risk of all-cause mortality compared to the general population (3). The majority of premature mortality in T2D is due to cardiovascular disease (CVD), the leading cause of death in T2D (3, 4). CVD includes disease related to pathology within the heart and vasculature including but not limited to coronary artery disease, heart failure, hypertension, cerebrovascular accidents and peripheral artery disease (5). One major modifiable risk factor related to cardiovascular mortality is cardiorespiratory fitness (CRF). CRF is a measure of the circulatory and respiratory systems’ ability to supply oxygen to skeletal muscle during sustained exercise (6). Maximum oxygen uptake during peak exercise (VO_2peak_) is the gold standard measure of CRF and is a strong predictor of all-cause mortality in T2D as well as many other disease states (7–9). Lower CRF is also predictive of short-term CVD events and increased risk of sudden cardiac death (10, 11). Physical inactivity, which results in decreased CRF, is an additional independent risk factor for all-cause mortality (12, 13). Fortunately, CRF can be improved through exercise (14, 15). In addition to the influence of exercise on CRF, and therefore mortality, exercise has also been shown to have beneficial effects on HbA1c, hypertension and body mass index (BMI) in type 2 diabetes (T2D) making it a cornerstone of treatment (16, 17). Similarly, exercise is beneficial to the general population and in people with prediabetes as it lowers the risk of developing T2D (18, 19). Major medical societies including the American Diabetes Association (ADA), American College of Cardiology (ACC), American Heart Association (AHA) and American College of Sports Medicine (ACSM) include exercise recommendations in their guidelines as a risk modifier to reduce CVD risk. It is recommended that all adults participate in 150 minutes or more per week of moderate intensity aerobic exercise, such as biking, dancing or brisk walking or 75 minutes or more per week of vigorous intensity exercise, such as running, singles tennis or swimming laps in addition to 2-3 sessions per week of resistance training exercise (20–22). Unfortunately, as noted, people with T2D exercise less than the general population and the majority do not meet this recommendation (23–27). Even when people with T2D participate in exercise programs, few maintain these changes long term (28, 29). The contributors to decreased exercise participation include the universal societal barriers to exercise, such as busy schedules and competing interests influencing motivation, in addition to those specific to people with T2D. Several qualitative studies have also explored reasons why people with T2D exercise less (30, 31). Answers reflect a wide variety of obstacles including physiological barriers such as “physical discomfort,” psychological barriers such as “lack of motivation,” social barriers like “lack of childcare” and environmental barriers including “weather” (30–32). In this narrative review, we will explore the physiological and societal frameworks behind these hurdles to better understand exercise patterns in those with T2D. We will also explore novel, evidenced-based solutions required to overcome each of these barriers to increase exercise for people with prediabetes and T2D (**Figure 1**).

## 2 Physiological barriers to exercise in T2D and potential solutions

### 2.1 Barrier: reduced cardiorespiratory fitness

Physical symptoms are among the most reported barriers to exercise in people with T2D (23–27, 30). The majority of people with T2D are sedentary with as many as 39% of patients with T2D reporting either “physical discomfort” or being “too tired” as their primary reason for not exercising at recommended levels (23–27, 30). However, there are other reasons why people with T2D might not exercise. One potential reason is that people with T2D have impaired peak exercise performance compared to activity and weight similar controls without diabetes, a deficit that appears to be more pronounced in women than men (33, 34). Specifically, on graded exercise testing, sedentary individuals with uncomplicated T2D displayed 24% less maximal walking time and 20% lower VO_2peak_ than age and activity similar controls without diabetes, regardless of glycemic control (35). In terms of sex differences, women with T2D had a greater deficit in VO_2peak_ versus women without diabetes, a 24% reduction, when compared to men with T2D versus men without diabetes, a 16% reduction (33). This limitation is particularly significant since lower CRF, measured by VO_2peak_, strongly correlates with increased cardiovascular mortality, and may explain the higher mortality rate in women with uncomplicated T2D compared to men with uncomplicated T2D (15). The mechanism by which this impairment occurs is complex and multifactorial. Physiological mechanisms of the impaired oxygen consumption are being explored and include a combination of metabolic, cardiac and peripheral factors including insulin resistance, mitochondrial dysfunction, vascular endothelial dysfunction and cardiac dysfunction (36). No single mechanism fully explains the deficit in CRF, but the sum of cardiac, skeletal muscle, circulatory and metabolic abnormalities of these impairments likely contribute to physical symptoms hindering exercise performance in people with T2D.

### 2.2 Barrier: greater perceived exertion

Greater perceived exertion is one of the most commonly cited reasons why people with T2D do not exercise (30). Women with T2D were compared to controls without diabetes to determine differences in perceived exertion (37). Rate of perceived exertion (RPE), a measure of exercise exertion, was measured in women with uncomplicated T2D by the self-reported Borg scale during submaximal bicycling exercise and compared to RPE in obese and lean women without diabetes. The group with T2D perceived higher rates of exertion compared to both obese and lean women without diabetes. In another study, RPE was recorded during every minute of maximal stress testing in people with T2D to determine if subjective perception of moderate exercise intensity correlated with target heart rate for moderate exercise intensity (38). Almost half of individuals were unable to accurately perceive moderate intensity exercise (as defined by heart rate) which further suggests limitations in perception of exertion during exercise in T2D. Those who were unable to appropriately identify moderate intensity exercise were more likely to be female, African American or Hispanic as well as have a higher BMI and lower fitness level. Increased perception of exercise exertion compared to people without diabetes remains a physiological barrier to exercise in T2D. This difference in perception is poorly understood and further research is needed to identify mechanisms to explain this phenomenon. It is possible that interventions, either pharmacological or otherwise, that decrease perceived exercise exertion could support greater engagement in exercise behaviors.

### 2.3 Solution: improving cardiorespiratory fitness through exercise training

If inherently reduced CRF is associated, at least in part, with the physical symptoms that are barriers to exercise in people with T2D, then increasing CRF can be a possible solution to increasing exercise participation. Increasing CRF can be challenging since the best way to accomplish this is through exercise training and/or increased physical activity. In a prospective cohort study of 72,624 individuals with a variety of CVD risk factors, including diabetes, high cholesterol, and hypertension among others, there was a strong association with self-reported physical activity and higher CRF (15). Furthermore, in a different study, women with uncomplicated T2D, who have lower CRF, overweight women without diabetes and lean women without diabetes underwent a 3-month exercise training intervention with pre and post exercise testing to see if CRF would improve with exercise training (14). The exercise intervention consisted of 60 minutes of supervised aerobic exercise 3 times per week to achieve 70-85% maximum heart rate for 3 months. After completing the exercise program, women with T2D showed 28% improvement in VO_2peak_ compared to 5% in overweight women without diabetes and 8% in lean women without diabetes (P<0.05 when T2D group compared to both control groups) representing the ability to modify CRF in T2D with exercise training. Women with T2D started with the lowest CRF and had the greatest increase in CRF with exercise, emphasizing the potential benefits of exercise training in T2D. The U-TURN trial provides an additional example of the benefits of exercise in T2D. Adults with T2D participated in a 12 month lifestyle intervention including 5-6 sessions per week of partially supervised aerobic and resistance exercise, which allowed for graduated autonomy over a period of 4 months and an individualized diet plan focused on macronutrient distribution. The participants in the lifestyle intervention had improved glycemic control compared to the group receiving standard of care alone (39). Participants were interviewed at completion of the study and at a 6-month interval. They stated their main motivators to maintain exercise were “improved fitness and physical form” and “improved well-being” (40). The problem remains that initiating exercise training to improve CRF requires the individual to participate in exercise. Overcoming inertia related to baseline low CRF requires creativity on the part of the individual and their coach or provider. Lessons can be taken from cardiac rehabilitation programs where step one is overcoming deconditioning prior to effective engagement in conditioning or exercise training programs (41). One term for initiating an exercise program in an inactive person is “conditioning” with a focus on gradually increasing activity with stretching, warm-up and cool down.

### 2.4 Solution: improving cardiorespiratory fitness through medications

Great effort and expense have gone into identifying potential pharmacological strategies to improve CRF. The search for exercise mimetics has been largely ineffective to date. However, some small physiological studies have demonstrated improvements in CRF with medications that have effects that might translate to improving exercise capacity (42, 43). Ideally medications or other strategies to decrease impairment in CRF could set the stage for encouraging individuals to incorporate exercise into daily life. For example, we and others have demonstrated that rosiglitazone, a thiazolidinedione, improves CRF in patients with T2D (42, 43). In 2005 participants with uncomplicated T2D were randomized to rosiglitazone 4mg/day or placebo (42). Exercise capacity, endothelial function and insulin sensitivity were measured at baseline and after 4 months of intervention. Compared to placebo rosiglitazone was associated with an improvement in VO_2peak_, endothelial function and insulin sensitivity (42). This phenomenon was corroborated in 2007 when a similar study also demonstrated an increase in VO_2peak_ and insulin sensitivity with rosiglitazone versus placebo (43). It’s important to note that increases in CRF are even greater with rosiglitazone plus exercise than either rosiglitazone or exercise alone (44). However, rosiglitazone is not in wide use at this time. Notably, the currently available thiazolidinedione, pioglitazone, also improves CRF (45).

To our knowledge other potential exercise mimetics have not shown a direct measured benefit in CRF in people, or have not been studied for this outcome, but may still target the mechanisms to ameliorate impaired exercise capacity. The large NIH multi-center study evaluating the molecular transducers of exercise (MoTrPAC) may inform targets for exercise mimetics since this study is examining the molecular basis of exercise (46).

In animals numerous agents have been shown to either increase VO_2peak_ or endurance but to date, compelling studies to support their use in humans are lacking (47, 48). The enzyme 5’ adenosine monophosphate-activated protein kinase (AMPK) is a critical bioenergetic sensor in the skeletal muscle stimulated and necessary for exercise training responses; many molecular *in vitro* and animal studies have explored AMPK activators as exercise mimetics (49, 50). Another example, glucagon-like peptide 1 (GLP 1) agonists, have known cardiovascular benefits and have been shown to improve skeletal and cardiac muscle insulin resistance through nitric oxide dependent endothelial dilation in animal and human studies (51–55). Dipeptidyl Peptidase-4 inhibitors, that work on the same incretin pathway as GLP 1 agonists, have been shown in rats to improve mitochondrial adaptation to exercise (56). Angiotensin II receptor antagonists and angiotensin-converting enzyme inhibitors ameliorate production of advanced glycation end products in animal models, which are pathologic in development of microvascular complications of T2D (57–61). L-arginine has also been demonstrated to improve endothelial dilation and therefore insulin sensitivity in human studies (62, 63). Searching for exercise mimetics is an exciting pursuit; although to date agents have not been discovered to fully mimic the systemic impact of exercise. Targeting and reversing various mechanisms of exercise impairment in T2D may lead to improved CRF and therefore fewer physical symptoms of exertion during exercise and improved exercise self-efficacy. Exercise in combination with agents that activate peroxisome proliferator-activated receptor (PPAR) delta have shown promise as combination therapy in animal studies (47). At this moment more investigation is needed in this area and interventions involving medications in combination with behavior change are most likely to enable durable improvements in CRF.

## 3 Psychological barriers to exercise in T2D and potential solutions

### 3.1 Barrier: motivation and self-efficacy

Psychological barriers also contribute to low exercise levels in people with T2D, which have been expressed in interviews as “too boring,” “dislike,” “negative past experiences,” “embarrassed” and “I just feel like giving up” (30, 40). These statements reflect feelings of poor motivation and low self-efficacy, or one’s belief in their ability to perform a behavior to achieve a specific goal (64). It is challenging to disentangle motivation per se from symptoms of depression, which are common in people with diabetes or diabetes distress which is emotional pain related to living with diabetes. Diabetes distress, which is specifically related to the demands of daily life and fear about short-and long-term consequences of diabetes, encumbers many people with diabetes and impacts self-efficacy (65). Considering diabetes distress and high risk for depression is essential when trying to positively impact diabetes self-efficacy.

Qualitative studies assessing barriers to exercise in people with T2D negatively describe low perceived motivation as “lack of will” (45.6-59%) and “lack of time” (20.3-31.5%) in addition to low self-efficacy as “lack of skills” (21%) (66, 67). Both depression and diabetes distress can exaggerate these barriers. Motivation and self-efficacy, influenced by diabetes distress, not only affect exercise, but also many self-management aspects of diabetes including diet, glucose monitoring and medication persistence. In one study, diabetes related distress was found to have a negative impact on self-care behaviors, such as exercise (68). In another study employing the Problem Areas in Diabetes Survey (PAID) to measure diabetes distress, there was also a significant decrease in diabetes self-care behaviors including exercise in those with higher diabetes distress scores (65). This is of particular interest since 36% of people with T2D experience diabetes related distress (69). A cross-sectional analysis of adults with T2D who completed a structured interview regarding barriers to exercise self-reported that feeling a lack of self-efficacy is strongly associated with suboptimal diabetes self-management behaviors. Specifically, in a sample of individuals with diabetes not at goal (HbA1c >7), higher self-efficacy scores in diet, performance of exercise, blood sugar testing and medical treatment were associated with higher ability to reach their goals in relation to diet, exercise, blood sugar testing and taking medications (70). One’s perception of their motivation and self-efficacy reflect components of self-regulatory behavior, a target for overcoming psychological barriers to exercise.

### 3.2 Solution: technology and self-regulatory behavior

Self-regulatory behavior is the ability to regulate behavior through self-monitoring, judgement of the behavior in relation to personal and environmental standards and appropriately adjusting behaviors to meet goals, and it is well studied (71). Self-regulatory behavior can be applied, often through technology, to strategically overcome psychological barriers to exercise in T2D (72). A meta-analysis of 30 reviews evaluating the most effective techniques for increasing physical activity found that interventions incorporating self-regulatory behavior strategies were associated with increased physical activity and weight loss (73). In one study of adults with prediabetes aimed at increasing and maintaining resistance training, additional action theory tests were conducted to determine which intermediary targets of behavior change were most successful at producing sustained increase in exercise. After 18 months maintenance of behavior change was most influenced by self-regulatory behavior. In another study, self-regulatory behaviors included reporting workouts online after completion, scheduling workouts in advance and participating in active problem solving to overcome barriers to attending workouts (74).

Many recent technological interventions, including smart phone applications, online coaching platforms and fitness technology can help increase physical activity, decrease sedentary time and promote weight loss (75, 76). The most impactful health technologies utilize self-regulatory behavior strategies (77, 78). A systematic review of 26 studies examining technology on the influence of objectively measured physical activity found that the most useful strategies to promote physical activity change included goal setting, real-time feedback, physical activity profiles and social support networking, all factors of self-regulatory behavior (77). In a similar review, also noting the impact of self-regulatory behavior, the authors recommend the next step in advancing fitness technologies is to incorporate cognitive behavior therapy focused on developing positive feelings towards exercise (78). Cognitive behavioral therapy has been incorporated into apps focused on beliefs and barriers surrounding various diabetes-related behaviors. In one randomized controlled trial, people with T2D using an app with cognitive behavioral therapy had significant reductions in HbA1c compared to placebo (79). More exercise specific research in people with T2D is needed to assess and augment the efficacy of cognitive behavioral therapy in fitness technology.

One popular method to incorporate self-regulatory behavior into daily life is through wearable activity trackers. Wearable activity trackers, often watches, harness aspects of self-regulatory behavior including goal setting, most often through monitoring steps per day, and feedback through real-time tracking of physical activity. A 2019 meta-analysis of 26 randomized controlled trials evaluated the impact of wearable activity trackers on physical activity in adults. Compared to nonactivity tracker interventions, participants with wearable activity trackers had a significant increase in daily step count, an average of 627 additional steps/day (80). This increase in physical activity with wearable activity trackers is consistent when studied in people with T2D (81, 82). In one study the use of wearable fitness trackers (FitBit©) in people with T2D showed a statistically significant increase in average weekly minutes of walking from 358 to 507 (p=0.04) (82). In a narrative interview examining influences on behavior change after a 12-month lifestyle intervention for people with T2D, including structured exercise with wearable fitness trackers, participants who maintained lifestyle changes stated they were motivated by feedback from their step tracking device. In another example of self-regulatory behavior, participants were also motivated by being able to reduce their diabetes medications, asserting “I see what I’m doing is working.” (40). Others when interviewed highlighted the importance of social accountability from the coaches and participants, leading to increased feelings of commitment and responsibility to the group. Social community and accountability are principal factors of self-regulatory behavior noted in many studies as a key influence on effectiveness (40, 73, 77, 78). In fact, one meta-analysis of interventions including both physical activity and diet in people at risk for diabetes reported that adding social support to interventions accounted for an additional 3.0kg of weight loss at 12 months compared to those without social support (73).

### 3.3 Solution: the role of physicians and healthcare teams

Physicians and healthcare team members influence exercise beliefs and behaviors of their patients (40) (23). The healthcare provider’s overall approach to diabetes self-management, education, and support greatly contributes to a tone of encouragement and motivation or judgement and blame. The ADA recommends healthcare providers engage in person-centered assessments to identify and address barriers encountered by patients (83). By using shared decision making and person-first, non-judgmental language, the healthcare provider can establish trust which in turn can be motivating for behavior change in people with diabetes (84). A cluster sampling of 48 general practitioners and their 369 patients with T2D demonstrated that the general practitioner’s level of perceived barriers to patient exercise correlated with the patient’s level of perceived barriers to exercise and low physical activity levels (23). Thinking of exercise as an individualized prescription and anticipating barriers in advance may help both physicians and patients achieve physical activity goals. Instead of counseling generally about 150 minutes of moderate-intensity exercise weekly, one method to individualize exercise prescriptions includes specifying frequency, intensity, time and type (FITT) (85). For example, a patient with T2D and knee osteoarthritis who works full time, could be prescribed moderate intensity swimming for 50 minutes, three days per week in anticipation of potential barriers of knee pain and time traveling to the gym daily. In addition to advice from physicians, several studies have shown the benefit of multidisciplinary teams improving physical activity among patients with T2D (86, 87). A randomized controlled trial measuring sustained 3-year change in physical activity in sedentary patients with T2D in Italy showed patients receiving regular counseling sessions by diabetologists and exercise specialists had higher levels of physical activity when compared to standard care general physician recommendations (86). Similarly in the US, a pragmatic pilot trial established feasibility and acceptable cost, reimbursable by the Medicare Chronic Disease Management Program, of a multidisciplinary program termed Be ACTIVE, which included physical activity tracking with FitBit©, six biweekly calls with a physical activity coach, and three primary care visits to ensure safety among other exercise education (87). This multidisciplinary intervention yielded improved Short Physical Performance Battery scores, which predict reduction in clinical falls, and a non-statistically significant increase in physical activity. Importantly patients appreciated support from their coach and accountability provided by the physical activity tracker (87). Frequent contact with healthcare professionals can encourage physical activity, even if only to reinforce previously implemented positive behavior change (40). A more general approach, such as the 150 min per week recommendation also remains of benefit, given that not all people with T2D may have access to more individualized recommendations.

### 3.4 Solution: Fogg behavior model

The Fogg Behavior Model, which incorporates elements of self-regulatory behavior, suggests behavior is a result of simultaneous motivation, prompting and ability (88). While the Fogg Behavior Model has not been formally studied in T2D, we examine the individual elements of the Fogg Behavior Model as they have been applied to overcome barriers to exercise in people with T2D (89). A study investigating the effect of motivation in patients with T2D on physical activity showed that higher self-motivation, measured qualitatively as less apathy, less dislike, more support and more knowledge, correlated with higher levels of physical activity and exercise (89). Motivation prompting behavior change is also closely tied with feedback and social accountability (40). Motivation combined with real-time prompts can further impact behavior. A feasibility study of a health application used “just in time” prompts to remind users to initiate runs, walks or strength exercises. With use of prompts users reported increased intrinsic motivation for exercise and increased perceived capability or self-efficacy to exercise (90). Self-efficacy is closely tied with ability, the final ingredient to the Fogg Behavior Model. While one single factor has not been identified to increase self-efficacy related to increased physical activity, a systematic review showed that interventions that utilized more behavior change techniques were associated with increased self-efficacy (91). Additional interventions to increase exercise in those with T2D designed to address all three elements of the Fogg Behavior Model including motivation, prompting and ability simultaneously are needed.

### 3.5 Solution: continuous glucose monitoring

The ADA Standards of Care 2023 describe the traditional use of continuous glucose monitors (CGMs). “Real-time continuous glucose monitoring should be offered for diabetes management in adults with diabetes on multiple daily injections or continuous subcutaneous insulin infusion who are capable of using the devices safely,” supported by randomized controlled trials with a level of evidence A (21). Additionally, there is expert consensus that use of glucose monitoring, not specifically CGMs, expands beyond just for those on intense insulin regimens. This consensus is reflected in the ADA Standard of Care statement, “Although blood glucose monitoring in individuals on noninsulin therapies has not consistently shown clinically significant reductions in A1c, it may be helpful when altering nutrition plan, physical activity, and/or medications in conjunction with a treatment adjustment program.” (21). Several studies have shown increased self-efficacy and increased exercise with the use of CGMs in patients with T2D, supporting their use as a self-discovery tool to overcome barriers to exercise in T2D (92–96). A review of CGM use and its impacts on lifestyle change in people with prediabetes and T2D describes increased physical activity, decreased calorie consumption and weight loss with CGM use (92). A pilot randomized controlled trial randomized 13 participants with prediabetes or T2D to an 8-week exercise intervention with or without CGMs as a real-time feedback tool (95). After the initial 8-weeks and at 1 month of follow up, the self-monitoring group utilizing CGMs demonstrated higher self-monitoring, higher self-efficacy to self-monitor and goal setting. They also had higher attendance rates of exercise programs and were more likely to register for additional exercise programs. Another randomized controlled trial of 52 adults with T2D evaluated self-efficacy counseling through incorporating feedback on CGM graphs during physical activity compared to traditional diabetes education that did not include CGM use (93). The group receiving self-efficacy counseling on CGM graphs had a significant increase in moderate activity as well as decreases in sedentary time, HbA1c and BMI. Similar results were reported in another randomized controlled trial that evaluated intermittent CGM use, 3 days per month, in people with T2D over a 3-month period (96). Compared to the control group, instructed to self-monitor blood glucose four times per week, the real-time CGM group had a significant increase in total exercise time per week together with reduction in total calorie intake, weight, BMI and HbA1c. While not a formal guideline, expert consensus, supported by several randomized controlled trials, find that CGMs are beneficial for people with T2D that are not using insulin, by providing real-time feedback that can lead to positive behavior change including increased exercise (97). While promising, cost may be a barrier to broader CGM use and medical coverage for CGM with most providers is currently limited to people using insulin therapy. More research on cost-effectiveness of CGM use to facilitate effective and durable behavior change is needed to determine efficacy of CGM use in a broader set of people with prediabetes and T2D.

### 3.6 Solution: pet ownership

Pet ownership, particularly dog ownership, promotes increased walking which has been associated with reduced risk for CVD regardless of diabetes status (98, 99). One solution for self-described feelings of low motivation for physical activity, often named as a barrier to physical activity by people with T2D, can thus include dog ownership. Health benefits such as increased physical activity associated with pet ownership are well studied. In one study, dog owners were 34% more likely to achieve at least 150 minutes of exercise per week and 69% more likely to do any leisure-time physical activity compared to non-dog owners (100). In 2013 the AHA released a scientific statement suggesting that pet ownership, particularly dog ownership and increased physical activity were associated with a reduction in CVD, although a causal relationship cannot be determined due to lack of randomized controlled trials. Based on their review of a large body of evidence, pet ownership to reduce risk of CVD is now a Class IIb recommendation, with Level of Evidence B (99). Following this recommendation, the National Health and Nutrition Examination Survey found mixed results that pet ownership of a dog or cat was associated with lower prevalence of systemic hypertension, but not other cardiovascular risk factors including diabetes, heart failure, coronary artery disease or stroke after adjustments compared to non-pet owners (98). More research is needed, ideally ethically conducted randomized controlled trials without risk of harm to animals, to further determine the impact of pet ownership on CVD risk in people with T2D. There was a clear increase in physical activity associated with pet ownership, recently exhibited by puppy acquisition during the COVID-19 pandemic in 2020 (101). If feasible, pet ownership can be considered one solution to increasing physical activity in people with T2D.

### 3.7 Barrier: mental health and T2D

The increased prevalence of mental health disorders such as depression and anxiety in people with T2D compared to the general population has a significant impact on motivation and self-efficacy (102). Women with T2D have a higher overall prevalence of depression than men with T2D (23.8% vs 12.8%) (103). However, the likelihood of depression is more greatly influenced by diabetes in men compared to women with T2D (OR 1.9 vs 1.3). Additionally, the increased risks of mental health disorders and diabetes are bidirectional. The diagnosis of diabetes increases the risk of developing depression and severe symptoms of depression. Likewise, depression increases the risk of the development of diabetes and its complications (104). While not well understood, this bidirectional relationship is likely multifactorial involving autonomic and neurohormonal dysregulation (104). Unfortunately, people with T2D who are experiencing symptoms of depression are much less likely to exercise or engage in other self-management behaviors required for ideal diabetes management (105, 106).

### 3.8 Solution: physical activity improves mental health in T2D

Mental health disorders such as depression and anxiety, which occur in higher rates in people with T2D compared to the general population, are a well-known barrier to exercise (105, 106). Paradoxically, exercise is associated with fewer symptoms of mental health disorders and improved quality of life in people with T2D (107–109). Unfortunately, this solution requires overcoming the inertia of initiating exercise in order to become beneficial. A systematic review evaluating the effect of exercise on mental health in people with T2D demonstrated some improvement in symptoms of depression, anxiety and emotional well-being with exercise, although it acknowledges better randomized controlled trials are needed for corroboration (110). In 2019, 60 participants with T2D were randomized to a 12-week exercise intervention versus no exercise in the control group to evaluate the effect of exercise on mental health. At the end of the 12-week period, the exercise intervention group had significant improvement in self-esteem and mental health as well as reduced anxiety and insomnia compared to the control group (111). Fewer studies are available on the impact of physical activity on anxiety in people with T2D. A meta-analysis of healthy adults without diabetes demonstrated that increasing physical activity reduces symptoms of anxiety (112). Interventions that were most successful to reduce symtpoms of anxiety included those targeting only physical activity (instead of more than one health behavior), supervised exercise, moderate-high intensity exercise (instead of low-intensity) and exercising at a fitness facility (rather than at home). More research is needed to determine if similar reductions in anxiety symptoms in response to physical activity are also present in people with T2D. Mental health benefits from exercise can be particularly helpful in older adults with and without T2D, as evidenced by several systematic reviews demonstrating decreased symptoms of depression in older adults (113–115). One study of older adults with T2D of Puerto Rican heritage, who experience higher rates of diabetes compared to non-Hispanic Whites (38% vs 23%) showed significantly improved mental health via lower geriatric depression scale scores after a 16-week resistance exercise training program (116, 117). Furthermore, multiple regression analysis of people with diabetes, who reported a moderate to low quality of life, found that the only self-management behavior that was predictive of quality of life was level of physical activity (118). This finding further emphasizes the importance of physical activity as a solution for people with T2D to overcome psychological barriers to exercise including mental health disorders. In contrast to depression and anxiety, exercise is not consistently beneficial for diabetes distress as one component of diabetes distress is concern for changes in blood glucose, high or low, in response to exercise. The majority of research on diabetes distress is reported in people with type 1 diabetes. Treatment strategies for diabetes distress include acknowledgment of distress as a normal response to the demands of diabetes, peer-support, cognitive therapy including emotional regulation training, workshops with psychologists, diabetes educators, providers and emotional specialists, as outlined in a recent interventional study (119). More research is warranted in this area for people with all types of diabetes.

## 4 Social and cultural barriers to exercise in T2D and potential solutions

### 4.1 Barrier: women with T2D experience social and cultural barriers to exercise

Lack of social support is another common barrier to exercise reported by 9-29% of people with T2D, a barrier more frequently reported by females, closely influenced by cultural norms and expectations (30, 66, 67, 120). A qualitative study investigating barriers to exercise in South Asians residing in the United Kingdom with T2D found people had a well-informed understanding of the importance of exercise, but of the 32 respondents only 7 had attempted to increase their physical activity, and of those 7, only 1 was female (121). Barriers such as childcare and domestic responsibilities, putting others before oneself, language and maintaining cultural and religious standards are among deterrents to exercise in women with T2D and at risk for T2D across the world (32, 67, 121–124). In a systematic review, women with a history of gestational diabetes, and therefore at risk for developing T2D, commonly reported children and domestic responsibilities as a reason why they do not exercise. They also reported guilt associated with “putting themselves first” which expresses cultural norms of the role of wife and mother within a family unit (32). Other qualitative reports have gone even further to suggest women who take time to exercise are “selfish.” (121) South Asian respondents state that “once a woman got married, she was expected to stay indoors, attending to domestic chores and responsibilities: women cannot go out” (121). In Qatar, traditional cultural values impair womens’ ability to exercise. Many families still do not permit women to go outside unaccompanied (124). Additionally, conservative dress can hinder opportunities for exercise. South Asian women with T2D describe that they are unable to go to gyms or swimming pools to exercise because of cultural expectations of conservative dress around the opposite sex (67, 121). Language is an additional barrier. Spanish speaking women with T2D were more likely to report social barriers to exercise than their English-speaking counterparts of similar ethnic background (122). An anecdote from qualitative interviews describes a non-English speaking woman who was injured while walking outside and was unable to ask for help. Since that time, she has been “too frightened to go out alone,” therefore hindering her ability to exercise (121). Inability to communicate is isolating and a vulnerability experienced by women with T2D. Even with a shared language, women cite isolation as a reason they do not exercise. In a study of low-income African Americans with T2D, no childcare and “I do not have anyone to exercise with,” were common social barriers to exercise (125). Gender-specific social and cultural barriers to exercise in T2D are alarming given the known excess morbidity and mortality of women compared to men with T2D (33, 126). Even more concerning is the lack of gender and sex-specific research and cardiovascular guidelines that are desperately needed to provide equitable culturally competent care for those with T2D (127, 128). More research is needed in this area.

### 4.2 Solution: social and culturally competent interventions

Improving physical activity in people with T2D requires thoughtful application of solutions across cultures to address specific cultural barriers. In women with children who have a history of gestational diabetes, and therefore a 7-fold increased risk of developing T2D, a systematic review revealed that interventions which accounted for childcare were most effective, surpassing techniques such as education and wearable fitness devices that have had success in other populations (32). Studies in socially and culturally conservative populations have found remarkable success with walking as a method of exercise for women (123, 129). Although, as previously noted, some families in culturally conservative Qatar still require women to be accompanied when leaving the home, there has been a national cultural shift towards more independence for women. As culture has shifted, more women are able to exercise through walking without being required to be supervised by a family member as this is a more culturally appropriate form of exercise rather than sport (124). In other cultures walking is simply preferred by women as the exercise of choice due to feasibility, safety and avoidance of gyms and swimming pools (123). Similar preferences for walking as physical activity have been shown in middle-aged American women, mostly due to accessibility (129). Children, although sometimes a barrier to exercise, can also be inspirations for exercise. Women with T2D who view themselves as role models for their children are more likely to participate in exercise (32). Additionally older women who have grown children, particularly daughters, who support their exercise habits find their children to be a positive social influence (124). Culturally competent interventions have been studied with variable success. A culturally competent intervention for low-income African Americans with T2D provided culturally tailored education on diet and physical activity in low-income African American communities, from African American educators and leaders. Outcomes included a decrease in HbA1c at 6 months, which was not sustained at 12 and 18 months. Additionally, there was no increase in physical activity with this intervention (130). Similarly, in Qatar, a culturally competent intervention addressing Arabic language, cultural health and food habits and exercise was administered to people with T2D in a randomized controlled trial. The intervention resulted in significant reductions in HbA1c, BMI and albumin/creatinine ratio. Physical activity changes were not measured as an outcome (131). Overall, more culturally competent interventions are needed to improve exercise for people, particularly women with T2D as well as other self-management behaviors required in T2D.

## 5 Environmental barriers to exercise in T2D and potential solutions

### 5.1 Barrier: neighborhood deprivation

Neighborhood deprivation defined as the lack of resources in an area influencing socioeconomic status including many social determinants of heath (SDOH) such as income, housing conditions, living environment, education and employment influences risk of developing T2D as well as impact glycemic control, blood pressure control and LDL among other measures of cardiovascular risk (132, 133). Such CVD risk factors are associated with low CRF and increased CVD mortality (15). Higher indices of neighborhood deprivation are also associated with increased risk of all-cause, cardiovascular and cancer related mortality (134). There is emerging evidence that the built environment, considered as referring to man-made conditions including urban planning, landscape, architecture and transportation development, also impacts T2D (135, 136). This interaction primarily occurs through available food environment and physical activity opportunities. Some barriers to physical activity named by people with T2D are directly tied to the built environment including “dangerous roads” and “transportation issues” (30, 137). More data is needed to better understand the influence of the built environment on T2D and exercise.

### 5.2 Barrier: climate change

“Weather” has been a commonly cited environmental barrier to physical activity by people with T2D (30). Weather and seasonal variations impact physical activity in all-comers. For example, people are less active when it rains and there is typically an abrupt increase in physical activity in the spring after a less active winter (138, 139). While some weather impacts may be reduced by the built environment, there are newer data that extreme weather influenced by climate change has further reduced physical activity levels (140, 141). One article reviewing impacts of climate change on human health and physical activity found consistent negative effects of air pollution, natural disasters and extreme temperatures on physical activity levels (140). These changes were seen the most in people with chronic disease, such as T2D and overweight/obesity and in the aging population (140). Further evidence of this impact was seen while measuring urban trail use in Austin, Texas. As anticipated, extreme temperatures were associated with decreased trail use and therefore reduced physical activity (141).

### 5.3 Solution: built environment and climate change

Walkability and open space have been associated with lower risk of developing T2D and lower overall prevalence of T2D, most likely through creating safe spaces for sustained increased physical activity (136). Designing built environments to encourage physical activity for all community members would include development of sidewalks, crosswalks, green space, playground equipment, fitness equipment, park renovations and transportation infrastructure (40). One systematic review revealed that improved infrastructure for walking, cycling and public transportation was associated with increased overall and transportation-dependent physical activity, in part owed to improved aesthetics and perceived safety (142). Specifically for older adults, a systematic review of 60 studies showed that those who lived in the top 25% of the greenest neighborhoods had a lower risk of developing T2D (143). Intentional design of the built neighborhood with the intent to maintain higher levels of physical activity for community members can in turn increase CRF, which at sustained levels has been shown to decrease incidence of T2D (144). There may be solutions that positively impact both physical activity and societal contribution to preventing climate change, such as decreasing use of fossil fuels via improving access to walking and cycling as means of transportation. More research on the intersection of climate change, physical activity, and health is needed.

## 6 Conclusion

The global prevalence of T2D is increasing and is associated with increased mortality and morbidity compared to the general population, mostly due to CVD. Low CRF is a strong modifiable risk factor for CVD that can be improved with exercise, making it a cornerstone of diabetes treatment. People with T2D exercise less than the general population and do not exercise at recommended levels. In this narrative review we explore traditional and novel barriers to exercise in those with T2D, including impaired peak exercise performance (an impairment more pronounced in women than men), greater perceived exertion during exercise, self-reported low perceptions of self-efficacy and motivation, mental health disorders, and lack of social and cultural support. Environmental barriers include neighborhood deprivation, lack of safe walkable space, and climate change. Potential unique evidence-based solutions to overcome these barriers include self-regulatory behavior strategies utilizing wearable fitness trackers, CGMs as a real-time feedback tool, and promoting thoughtfully built environments to increase green space for physical activity. We discussed the benefits of exercise to increase CRF and potentially reduce uncomfortable physical symptoms that are often barriers to exercise. Additionally, we discussed how exercise can decrease symptoms of depression to help promote sustained exercise behaviors. Continued research to augment physical activity in T2D is needed, especially in women who have unique barriers to exercise and a higher mortality rate than men with T2D. Interventions should focus on creating sustainable increases in exercise that target improvements in CRF to ultimately improve global mortality from T2D.

## Author contributions

All authors listed have made a substantial, direct, and intellectual contribution to the work and approved it for publication.
